# P-1561. Use of BacterioScan 216Dx to Reduce Antibiotic Use for Suspected Urinary Tract Infection in the Emergency Department

**DOI:** 10.1093/ofid/ofae631.1728

**Published:** 2025-01-29

**Authors:** Kelly Gao, Vishak H Kumar, Giovanni Divinagracia, Keith S Kaye, Ahmed Abdul Azim, Thomas Kirn, Bert M Berla

**Affiliations:** Robert Wood Johnson Medical School, New York, New York; Rutgers Robert Wood Johnson, Holland, Pennsylvania; Rutgers Robertwood Johnson Medical School, Matawan, New Jersey; Rutgers Robert Wood Johnson Medical School, New Brunswick, NJ; Rutgers Robert Wood Johnson Medical School, New Brunswick, NJ; Rutgers Robert Wood Johnson Medical School, New Brunswick, NJ; Bacterioscan, Inc, St. Louis, Missouri

## Abstract

**Background:**

Urinary tract infections (UTI) are often empirically treated in the emergency department (ED) before urine culture (UCx) results are available. 20-40% of suspected UTIs yield negative UCx and therefore are, by definition, not UTIs. BacterioScan 216Dx is an FDA cleared device that allows for point of care analysis of urine samples for bacteria in 3 hours as opposed to UCx which can take up to 48 hours. The BacterioScan system uses the trend of optical density of a urine sample mixed with bacterial growth media to identify UTI positive samples containing ≥50,000 colony forming units (CFU)/mL of a uropathogen. We describe the potential impact of a negative BacterioScan test result on antibiotic use prior to UCx results becoming available.

Table

**Figure:**
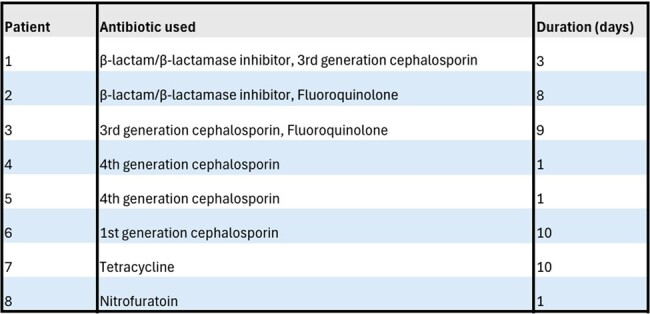

Urinalysis Positive, BacterioScan Negative, and Urine Culture Negative Patients Treated for Urinary Tract Infection: Antibiotics Used and Duration

**Methods:**

We performed a retrospective review of ED patients from 12/1/2023 to 4/8/2024 with a positive UA (WBC >5 cells/hpf or leukocyte esterase or nitrite +) that reflexed to culture. Concurrently, the samples were tested using BacterioScan, but results were not released to treating clinicians. BacterioScan and UCx results were recorded along with patient demographics, antibiotics received, and duration of treatment. This analysis focused on UA positive, BacterioScan negative patients.

**Results:**

600 patients had a positive UA that reflexed to culture. BacterioScan was negative in 100 of these patients. These patients had a mean age of 47.67 (SD 20.67). 66% were female. 36% identified as White, 16% African American, 4% Asian, and 44% other/prefer not to respond. 33% identified as Hispanic.

83 of 100 patients were Bacterioscan and UCx negative. Of these 83 patients, 8 (9.64%) were treated with median antibiotic duration of 5.5 days (Table).

17 patients had Bacterioscan negative/UCx positive results.13 of 17 had a mix of different bacterial species with CFUs ranging from < 10,000 to 50,000. 4 UCx were positive for potential pathogens- *Streptococcus agalactiae* (2), *Proteus mirabilis*, and *Klebsiella pneumoniae*. Of these 4, 3 had < 50,000 CFU and 1 had ≥100,000 CFU.

**Conclusion:**

Appoximately 10% of patients of patients with UA positive/BacterioScan negative/UCx negative results could have avoided antibiotic treatment if BacterioScan results had been available to clinicians in real time. In urgent care settings, BacterioScan can help reduce unnecessary antibiotic use.

**Disclosures:**

**Keith S. Kaye, MD, MPH**, Allecra: Advisor/Consultant|CARB-X: Advisor/Consultant|GSK: Advisor/Consultant|Merck: Advisor/Consultant|Shionogi: Advisor/Consultant|Spero: Advisor/Consultant **Thomas Kirn, MD PhD**, Selux Diagnostics: Advisor/Consultant|Selux Diagnostics: Honoraria **Bert M. Berla, PhD**, Bacterioscan, Inc.: Ownership Interest|Bacterioscan, Inc.: Stocks/Bonds (Private Company)

